# A CBT-based training module for UK health visitors who support parents with excessively crying babies: development and initial evaluation

**DOI:** 10.1017/S1463423624000082

**Published:** 2024-04-19

**Authors:** Ian St James-Roberts, Sarah Griffiths, Maggie Watson, Charlotte White, Jayne Brown

**Affiliations:** 1 Thomas Coram Research Unit, UCL Institute of Education, University College London, London, UK; 2 The Leicester School of Nursing, Midwifery and Paramedicine, De Montfort University, Leicester, UK; 3 Research Department of Clinical, Educational and Health Psychology, University College London, London, UK; 4 The Surviving Crying Study, Institute of Health and Allied Professions, Nottingham Trent University, Nottingham, UK; 5 Centre for Public and Psychosocial Health Research, Institute of Health and Allied Professions, Nottingham Trent University, Nottingham, UK

**Keywords:** cognitive behaviour therapy, health visiting, infant crying, parenting support, primary healthcare

## Abstract

**Background:**

Parents report that around 20% of infants cry a lot without apparent reason during the first four postnatal months. This crying can trigger parental depression, breastfeeding cessation, overfeeding, impaired parent–child relationships and child development, and infant abuse. The Surviving Crying (SC) cognitive behaviour therapy (CBT)-based materials were developed in earlier research to improve the coping, wellbeing and mental health of parents who judge their infant to be crying excessively.

**Aim:**

This study set out to:

- develop a health visitor (HV) training module based on the SC materials, tailored to fit health visiting;
- assess whether HVs could deliver a SC-based service successfully;
- confirm whether parents gained similar benefits to those in the earlier study;
- prepare for a controlled trial of the SC-based service.

**Methods:**

A training module was developed to enable HVs to deliver the SC materials, much of it provided online. Ten HVs took the training module (‘SC HVs’). They and the Institute of Health Visiting provided feedback to refine it. SC HV delivery of the CBT sessions to parents with excessively crying babies was assessed using a standardised test. Parental wellbeing was measured using validated questionnaires. Parents and SC HVs evaluated the effectiveness of the SC service using questionnaires or interviews.

**Findings:**

The study produced the intended training module. Most SC HVs completed the training, and 50% delivered the SC-based service successfully. Both training and delivery were disrupted by the Covid-19 pandemic, illness and work pressures. Replicating earlier findings: most parents’ anxiety and depression scores declined substantially after receiving the SC service; improvements in parents’ confidence, frustration and sleep were found; and all parents and the SC HVs interviewed found the SC service useful and agreed it should be included in the National Health Service. A controlled trial of the resulting SC service is underway.

## Introduction

Parents report that around 20% of infants cry for prolonged periods without apparent reason during the first four postnatal months (Douglas and Hill, 2011; Wolke et al., 2017). There is objective evidence that they are often correct in reporting prolonged crying (St James-Roberts *et al.*, 1996). Based on 2021 figures, this affects some 125 000 families in England and Wales per year (Office for National Statistics, 2022). Traditionally, this phenomenon was known as ‘colic’, implying that the crying is due to gastrointestinal disorder and pain. Despite many studies, however, the crying remains unexplained, and there is no reliable way to prevent or resolve it (Sung *et al.*, 2014; St James-Roberts, 2018).

Recently, the need to understand how parents cope with this crying, and subsequent outcomes, has become apparent. Many parents worry that the crying is a sign their baby is unwell (Barr *et al.*, 2000). However, the impact on parental emotions and behaviours depends on their resources. Anxiety and emotional arousal influence how parents interpret infant crying (Pearson *et al.*, 2010; Laurent and Ablow, 2012), and this can precipitate parental frustration (Fujiwara *et al.*, 2011) and depression (Petzoldt, 2018). Other adverse outcomes include premature termination of breastfeeding (Howard *et al.*, 2006), overfeeding (Stifter *et al.*, 2011), impaired parent–child relationships and child development (Papousek *et al.*, 2001; Smarius *et al.*, 2016), and infant abuse in rare cases (Barr *et al.*, 2006; Narang *et al.,* 2020).

Against this background, the lack of evidence-based National Health Service (NHS) provisions for supporting parents in managing infant crying is striking. Instead, parents turn to popular media, which give conflicting advice (Catherine *et al.*, 2008), or take infants to doctors or hospitals (Freedman *et al.*, 2009), adding to already substantial NHS costs (Morris *et al.*, 2001). In Canada, a support programme for parents reduced hospital admissions for infant abusive head trauma and proved cost-effective in the long term (Barr *et al.*, 2018; Beaulieu *et al.*, 2019).

With the above findings in mind, the initial ‘Surviving Crying’ (SC) study was funded by the National Institute for Health and Care Research (NIHR) to develop materials that support UK parents who are concerned about infant crying they judge to be excessive. The materials and detailed results from that initial SC study were reported in three publications (Powell *et al.*, 2018; Bamber *et al.,* 2019; St James-Roberts *et al.,* 2019). The materials included a cognitive behaviour therapy (CBT)-based website, booklet based on the website and short programme of CBT-based face-to-face sessions with a qualified practitioner. Following the National Institute for Health and Care Excellence guidelines (NICE, 2011), the website and booklet were intended to be inexpensive and available to all parents – the CBT sessions for families with greater need. CBT is a manualised psychological therapy widely used by mental health services and recommended by NICE as a treatment for mild to moderate depression (NICE, 2011) and for supporting parents’ mental health during the perinatal period (NICE, 2014).

To evaluate the SC materials, 52 parents with excessively crying infants were referred to the initial SC study team by NHS health visitors (HVs). After informed consenting, these parents completed standardised measures before receiving the SC materials (baseline) and afterwards. Almost all the parents accessed the website or booklet; half accessed the CBT sessions. At baseline, 30 of 52 parents met clinical criteria for depression, halving to 15 after receiving the SC package (Powell *et al.,* 2018). In regression analyses, these improvements were not explained by changes in infant crying (St James-Roberts *et al.*, 2019). Reductions occurred in numbers of parents reporting the crying to be a large or severe problem (from 28 to 3 parents) or feeling very or extremely frustrated by the crying (from 31 to 1 parent). Other findings included improvements in parents’ confidence, knowledge of infant crying, sleep and reduced NHS contacts (Bamber *et al.*, 2019; St James-Roberts *et al.*, 2019). All 52 parents and 85% of 50 HVs agreed the package should be included in the NHS; 94% of the HVs wanted materials of this kind included in HV training (Bamber *et al.*, 2019).

For the initial SC study, researchers delivered the website and booklet, and a psychologist delivered the CBT sessions. However, because HVs and allied professionals provide primary healthcare support for all UK parents and infants, HVs were closely involved in the initial SC study, and the SC materials were designed to be compatible with statutory HV services.

### Aims and objectives

The purpose of the new study reported here was to develop the SC materials into a training module tailored to HV needs and to assess whether HVs could deliver the resulting CBT-based SC service under routine NHS conditions. Although there is evidence that nurses, including HVs, can deliver CBT sessions like those in the SC package (Morrell *et al.*, 2009; Chowdhary *et al.*, 2014), HV services have experienced recent cuts and reorganisations, questioning whether HVs can deliver the SC materials in practice. Health visiting offers a promising way to deliver these supports as a primary healthcare service, but delivery effectiveness has to be demonstrated.

The objectives of the current study were as follows:

- To work collaboratively with HVs to develop a training module based on the SC materials and tailored to fit their needs.
- To assess whether the module can be used to train HVs in how to deliver the CBT-based support sessions and other materials and whether HVs can deliver the SC CBT-based support sessions successfully under routine NHS conditions.
- To learn whether parents involved gain similar benefits to those in the initial SC study.
- To provide the materials needed for a controlled trial of the SC service and consider whether that trial is warranted.

## Methods

### Development of a CBT-based training module for SC HVs

The training of SC HVs needed to be suitable for professionals with busy working schedules. Hybrid or ‘blended learning’ methods are well suited for this purpose (Horn and Staker, 2014). These combine online and classroom delivery of materials, allowing students to move at their own pace using materials available at all times. Following discussions with the Institute of Health Visiting (iHV, the professional body for UK health visiting) and senior HV collaborators in Lincolnshire and Sheffield areas of England, iHV specialist training modules on other topics provided a blueprint for the training. The research team prepared draft training materials based on the initial SC study. These were reviewed and revised until they were agreed to be suitable. The resulting module was designed to contain six main elements:

1. A Practitioner’s Manual providing step-by-step guidance on how to deliver the CBT-based sessions to parents. Supported by handouts for parents and other documents, the Manual provided the backbone for the training module and session record-keeping. Each session was designed to include two main parts: history taking about the infant’s crying, its impact on the parent, and any steps needed to check infant health and use of CBT-based methods to support parents in developing coping skills to manage their emotions, cognitions, and behaviours.
2. A day (6 h) of lectures to introduce the evidence about infant crying, its impact on parents, and CBT rationales and methods underlying the training module, followed by a group discussion of the lecture materials, all delivered online and facilitated by the research team. The lectures by two authors (MW and ISJR) were video-recorded, to provide a permanent resource that trainees could access at any time. The publications for the research cited in the lectures were also available online.
3. A one-day (6 h) Masterclass Workshop, designed to practice delivering the CBT sessions, held in person approximately one week after the lectures. Each part included a CBT trainer demonstration of how to deliver a session, followed by SC HV practice using role-play in subgroups to develop skills. The two trainers (MW and CW) were both CBT-qualified clinical psychologists. The topics covered were history taking and formulation, use of cognitive-focused CBT methods, and use of behaviour-focused CBT methods.
4. A half-day (3 h) Refresher Workshop, held in person or online about a week later, allowing SC HVs further practice in session delivery. Like the Masterclass, role-plays guided by written scripts and example records for a parent and infant were used to structure the practice sessions, with SC HVs rotating roles as a parent, an SC HV delivering this session, and an observer, followed by a question-and-answer session with the CBT trainers.
5. Supervision meetings, held online for 1 h per fortnight, to provide further guidance and support. Members of the research team facilitated the meetings, attended by SC HVs plus a CBT trainer where requested. Each meeting followed a pre-circulated agenda, generated collectively by SC HVs and trainers. Recordings of these meetings were sent to all SC HVs.
6. A standardised assessment of the SC HVs’ skills in delivering CBT-based support to parents, the Assessment of Core CBT Skills (ACCS, Muse *et al.*, 2017). This required SC HVs to submit a minimum of one audio recording of a mid-therapy session with a parent, and an accompanying cover sheet, for assessment by the CBT trainers. Each submission was assessed independently by both trainers. SC HVs who passed the assessment were awarded certificates confirming they had demonstrated good basic (level 2) CBT skills in supporting parents with an infant who cries excessively.

### Recruitment of SC and Regular HVs

Following contacts with HV services in Lincolnshire, England, 10 HVs provided informed consent to take part in piloting the training module (SC HVs). In addition, 96 Full-Time-Equivalent Lincolnshire HVs who did not receive the SC training were invited to briefings to learn about the study (Regular HVs). By agreeing to take part, they consented (1) to inform parents who were worried that their infant’s crying was excessive about the study and (2) to forward the contacts to parents who agreed to the research team. They provided HV services as usual but were not asked to take any other part in the study.

### Recruitment of parents with excessively crying babies

Parents in the Lincolnshire areas involved were routinely mailed a ‘new-birth pack’ containing information about services and resources. A letter informing them about the study was included in this pack. If a parent expressed concern about excessive crying in an infant up to 20 weeks of age, their Regular HV asked whether they would like to learn more about the study. Where parents gave written permission, their contact details were forwarded to the research team. A team member then made contact and offered to visit the parent at home to explain the study fully. If parents agreed, the researcher visited and sought parental written informed consent to take part. The researcher then completed baseline assessments of participants and provided a copy of the SC booklet and/or website access, according to the parent’s choice. Where parents chose to have the CBT-based sessions, the SC HV visited the parent at home to begin delivering them. Based on the initial SC study, most parents were expected to choose two to four sessions, each lasting approximately 1 h (Bamber *et al.*, 2019). Infants with a diagnosed medical condition were excluded from the study, as were infants older than 20 weeks because of evidence that they may have more serious disturbances (Wolke *et al.*, 2009).

### Baseline and outcome assessments

Table 1 lists the baseline questionnaires used, all of which proved effective in the initial SC study (Powell *et al.*, 2018). Except for the demographic questionnaire, these were repeated six to eight weeks later, after the SC intervention had been delivered (outcome measures). The parental evaluation of the SC materials (Bamber *et al.*, 2019) was also completed at the outcome stage. The SC HVs were interviewed to learn their views about the training module and sessions with parents. They were given individual feedback by the trainers on the ACCS assessment of their core CBT skills.

**Table 1.** Baseline and outcome measures

Name of measure	Content measured	Original reference
Demographic Information Questionnaire (baseline only)	Parental social, ethnic, educational and employment background	St James-Roberts *et al.*, 2019
Edinburgh Postnatal Depression Scale	Parental depression	Cox *et al.,* 1987
GAD-7 Anxiety Scale	Parental anxiety	Spitzer *et al.*, 2006
Sleep adequacy: 2 items from Pittsburgh Sleep Quality Index	Parental sleep quantity and quality	Hiscock *et al.*, 2014
Parenting Confidence Questionnaire	Maternal or paternal confidence in parenting	Badr, 2005 (originally called Maternal Confidence Questionnaire)
About your baby’s crying, feeding and health	Infant health, behaviour and remedies used to manage them since birth	St James-Roberts *et al.*, 2019
Crying Patterns Questionnaire	Infant crying amounts and daily patterns in the last week	St James-Roberts and Halil, 1991
Parental evaluation of the SC materials (outcome only)	Parents’ ratings of the SC materials they received	St James-Roberts *et al.*, 2019

GAD-7 = Generalised Anxiety Disorder; SC = Surviving Crying.

### Safeguarding

A safeguarding protocol and checklist to protect parents and infants in the initial SC study were developed by that study’s paediatrician, Leicester Partnership NHS Trust Safeguarding Officer, HVs and the research team (St James-Roberts *et al.*, 2019). This was reviewed and adopted by Lincolnshire Safeguarding staff and senior HVs for this study.

## Results

### Data analysis

This study was not designed to have groups large enough to allow tests of the statistical significance of findings. Instead, data analysis was guided by two principles. The first was the need for replication of findings, designed to provide evidence that findings are reliable, and widely considered a key requirement for scientific research (Dennis and Valacich, 2015). Reflecting this principle, the descriptive figures from this study are reported in relation to those from the separate, larger groups of parents (*n* = 52) and HVs (*n* = 50) assessed using the same methods in the initial SC study. The question addressed is whether the figures at baseline and outcome, and improvements between baseline and outcome, were consistent across the two studies, making it improbable that the findings reported here were due to chance. The second principle was the need for cost-effectiveness in deploying participants and resources. Rather than providing a stand-alone test of an SC-based service, this interim study was designed to prepare materials and methods for a controlled trial with large enough groups to allow full statistical and health economic data analysis.

### SC HV participation and acquisition of core CBT skills

Table 2 summarises these findings. The training and CBT-based sessions and skills assessment coincided with the Covid-19 pandemic. SC HVs, parents and researchers became ill; SC HVs were reassigned to deliver vaccinations or to other HV teams to replace unwell colleagues. Although the study provided funding to cover the time needed for their training and sessions with parents, this did not lead to direct SC HV support. The training had to be fitted into their busy workloads, already disrupted by the pandemic. Two became ill and withdrew from the study almost immediately, with two more withdrawing by the supervision stage because of workload pressures. Of the six who completed the training and supervision, five passed the ACCS assessment of CBT skills successfully. For unsuccessful trainees, a second ACCS assessment with a new recording and parent after further supervision was allowed. The trainers expected this SC HV to qualify on a second attempt, but recruitment of parents ended before this could be implemented.

**Table 2.** Rates of SC HV participation in the SC training module

No. SC HVs enrolled in the training module	No. took part in Day 1 lectures	No. took part in Masterclass Workshop	No. took part in Refresher Workshop	No. took part in supervision sessions	No. parents seen per SC HV	Mean (SD) no. CBT sessions delivered per parent	No. SC HVs took (passed) ACCS assessment of CBT skills
10	10	9 (1 unwell)	8 (2 unwell)	8 (2 withdrawn)	Range 1–3	2.53 (0.77)	6 (5)

SC = Surviving Crying; HV = health visitor; CBT = cognitive behaviour therapy; ACCS = Assessment of Core CBT Skills.

### Baseline descriptive figures for parents and infants

Twenty parents took part in baseline assessments and provided data. Their demographic and other descriptive figures are in Table 3. All were mothers. Fathers and other primary caregivers were eligible to take part, but none did so.

**Table 3.** Demographic and other descriptive figures for parents and infants in the study (*n* = 20 parents and infants)

Parental descriptives							
Mean (SD) age in years at baseline 29.4 (6.75)	Ethnic group: no. (%) WhiteEng/Welsh/Scottish/N Irish/British: 18 (90) Other White background: 2 (10)	Education: no. (%) GCSE O level/NVQ: 16 (80) GCSE A level: 11 (55) Degree or Postgrad: 7 (35)	Employment: no. (%) Mat. leave from full-time employment: 10 (50) Not in paid employment: 8 (40) Mat. leave from part-time employment: 1 (5) Student 1 (5)	Living arrangements: no. (%) Married/living with partner: 15 (75) Living with parents or friends: 1 (5) Living alone supported by partner: 2 (10) Single parent living alone: 2 (10)	Complications in most recent pregnancy: no. (%) 13 (65)	Complications in most recent labour or childbirth: no. (%) 10 (50)	Parent seen by GP for stress/anxiety/psychological wellbeing concerns: no. (%) 6 (30)
Infant descriptives							
Sex no. (%) Girl: 12 (60) Boy: 8 (40)	Birth order 1st born 8 2nd born 8 3rd born 4	Mean (SD) total hours of crying/24 h 8.06 (3.87)	Mean (SD) age in weeks when excessive crying started 2.87 (1.99)	Mean (SD) age in weeks at baseline 9.27 (2.83)	Feeding method when excessive crying started: no. (%) Breast milk: 9 (45) Formula milk: 11 (55%) Breast + formula milk: 3 (15)	Infant health concerns since birth: no. (%) Fever symptoms: 1 (5) Other symptoms unwell: 4 (20) Feeding problems: 7 (35) Poor weight gain: 2 (10) Regurgitating feeds: 12 (60)	Remedies to resolve the excessive crying: no. (%) parents/remedy HV advice at home visit: 18 (90) HV advice at clinic visit: 11 (55) HV advice by phone: 12 (60) GP advice at clinic visit: 12 (60) Changed feeding formula: 12 (60) Colic drops: 18 (90) Other crying remedy: 12 (60)

HV = health visitors.

All the parents were White and most were British. Typically, they received education up to GCSE/O level/NVQ level 2 and 11 to A level. Most were on maternity leave or not in paid employment and were married or living with a partner. Although the small numbers require careful interpretation, the finding that half or more reported complications during pregnancy and/or childbirth is consistent with the evidence from the initial SC study (St James-Roberts *et al.*, 2019). This appears a high percentage, although the lack of applicable reference figures prevents a firm conclusion. Six parents (30%) had seen their GP for stress or anxiety. This too is consistent with previous findings (Petzoldt, 2018; St James-Roberts *et al.*, 2019).

The mean infant age at onset of excessive infant crying reported by mothers was 2.87 weeks, while it was 9.27 weeks when the study’s baseline assessments were obtained. Some of this interval may have been linked to Covid-19, but the mean infant baseline assessment age in the initial, pre-Covid-19, SC study was 9.6 weeks, suggesting that some parents delay contacting HVs about excessive infant crying (Powell *et al.*, 2018).

Twelve infants were second or third born, echoing the evidence that the crying is not due to parental inexperience (St James-Roberts and Halil, 1991). The infants were more or less equally likely to be fed breast or formula milk. Few were unwell or had feeding problems or poor weight gain. Most (60%) regurgitated feeds, but this is common among infants at this age (Di Lorenzo, 2013). These findings too are consistent with the initial SC study (Powell *et al.*, 2018).

Most parents had tried remedies to resolve their infant’s crying, including HV and GP contacts, changing infant feeding formula, colic drops and a variety of other remedies (Table 3), adding to evidence that excessive infant crying is expensive for the NHS and the families involved (Morris *et al.*, 2001).

### Outcome compared to baseline measures

Twelve of the 20 parents with baseline data provided outcome assessments. The attrition was largely due to Covid-19-related infections, which made parents ill and disrupted HV services. Added to the small numbers, this attrition emphasises the need to interpret the findings cautiously. Table 4 lists the findings.

**Table 4.** Baseline (*n* = 20) and outcome (*n* = 12) measures for parents in this study

Depression: EPDS mean (SD) scores	Anxiety GAD-7 mean (SD) scores	Parenting confidence mean (SD) total scores	No. (%) parents finding infant crying to be a problem	How frustrated did parents feel about infant crying: no. (%)	Mean (SD) hours of parental sleep at night	Sleep quality rating score: no. (%) parents
Baseline 10.35 (5.06) Outcome 6.75 (3.22)	Baseline 7.1 (5.38) Outcome 3.92 (3.20)	Baseline 58.65 (7.32) Outcome 63.17 (4.88)	A severe problem Baseline 9 (45) Outcome 0 (0) A large but not severe problem Baseline 8 (40) Outcome 2 (17) A moderate problem Baseline 3 (15) Outcome 1 (8) A minor problem Baseline 0 (0) Outcome 4 (33) Not a problem Baseline 0 (0) Outcome 5 (42)	Extremely Baseline 9 (45) Outcome 0 (0) Very Baseline 3 (15) Outcome 1 (8) Moderately Baseline 4 (20) Outcome 3 (25) A little Baseline 3 (15) Outcome 1 (8) Not at all Baseline 1 (5) Outcome 7 (58)	Baseline 5.05 (1.61) Outcome 6.83 (1.93)	Very good Baseline 1 (5) Outcome 4 (33) Fairly good Baseline 7 (35) Outcome 4 (33) Fairly bad Baseline 4 (20) Outcome 4 (33) Very bad Baseline 8 (40) Outcome 0 (0)

EPDS = Edinburgh Postnatal Depression Scale; GAD-7 = Generalised Anxiety Disorder.

All 12 parents received the CBT-based sessions, receiving a mean (SD) of 2.53 (0.77) sessions per parent. This take-up rate was higher than in the initial SC study, where just half the parents chose to receive the CBT sessions. This may reflect a greater need during the pandemic, more enthusiastic endorsement of these sessions by this study’s Regular HVs, or parental variables. Nine also accessed the website and 11 read the booklet.

#### Parental depression

An Edinburgh Postnatal Depression Scale (EPDS) score of 10 or above is recommended as evidence of likely depression in community samples (Wisner *et al.*, 2002). At baseline, the parents’ mean score of 10.35 exceeded this criterion. Using this cut-off of ≥ 10, 12 of 20 (60% of) parents met the community screening criterion for depression at baseline. Using the recommended EPDS cut-off score of ≥ 13 (Levis *et al.*, 2020), 9 of 20 screened positively for clinical depression at baseline. At outcome, the mean score lowered to 6.75. Only one parent scored 10 or more, retaining her score of 13.

The initial SC study (Powell *et al.*, 2018) mean/SD EPDS scores at baseline (10.8/4.9) and outcome (7.10/4.5) were very similar to this study’s scores. Similarly, 30 of 52 (53% of) parents in the initial SC study scored 10 or more at baseline, while half (15) of them reduced their score to below 10 at outcome. However, 29% of parents in the initial SC study scored ≥ 10 at outcome, compared with one parent in this study. That is, substantial reductions in the numbers of parents with depression symptoms occurred in both studies, but more parents received normal EPDS scores at outcome in this study than the initial SC study.

#### Parental anxiety

Using the Generalized Anxiety Disorder (GAD-7) scale, a total score ≥ 5 signifies mild anxiety, ≥ 10 moderate anxiety and ≥ 15 severe anxiety (Spitzer *et al.*, 2006). The mean baseline score of 7.1 found here indicates mild anxiety in these parents (Table 4).

At baseline, 8 of 20 had mild, 2 moderate and 3 severe anxiety. At outcome, the mean score (3.92/3.20) reduced to below the threshold for mild anxiety. Four parents were mildly anxious at outcome, compared with 13 at baseline. One parent remained moderately anxious, compared with eight moderately or severely anxious at baseline. These findings, too, are promising and consistent with the initial SC study results in a larger and more complete dataset (Powell *et al.*, 2018). In that study, the mean/SD GAD-7 scores at baseline and outcome were 6.47/4.90 and 3.9/3.8, while the number of mildly, moderately and severely anxious parents all reduced (from 16 to 12, 11 to 2 and 4 to 2, respectively).

#### Parenting confidence

The Parenting Confidence Questionnaire mean total score increased from 58 at baseline to 63 at outcome (Table 4). This increase of five points resembles the four-point improvement (from 56 to 60) found by Powell *et al.*, (2018).

#### Parents’ judgements that their baby’s crying is a problem, frustration because of the crying and sleep

As Table 4 shows, these indices all improved at outcome, relative to the parents’ baseline measures, as in the initial SC study (Powell *et al.*, 2018; Bamber *et al.*, 2019). In that study, parental sleep per 24 h increased by 1 h 6 min per 24 h, the number of parents reporting crying to be a large or severe problem reduced from 50% to 6%, and the number of parents very or extremely frustrated by their baby’s crying reduced from 55% to 2%.

### Parental and SC HV opinions of the CBT and other components of the SC service

The CBT-based sessions were considered very useful by 11 of 12 and useful by 1 parent; 11 strongly agreed and one agreed that the sessions should be available in the NHS. All 9 parents who accessed the SC website and 10 of 11 who read the booklet found them useful or very useful. All 9 parents who visited the website, and 10 of 11 who read the booklet, agreed they should be available in the NHS. These findings closely resemble those from the initial SC study (Bamber *et al.*, 2019), where all the parents found the SC website, booklet and CBT-based sessions useful or very useful and agreed that they should be included in the NHS.

Due to their workload pressures, we did not collect quantitative outcome data from the SC HVs. Instead, they were offered the option of an online semi-structured interview, which was video-recorded with their permission. Four took part. All four had or would recommend the CBT training to colleagues and agreed that the CBT training had been valuable for them, helpful for parents, and should become part of HV training and the NHS. They also gave constructive feedback on areas for improvement, largely in terms of logistics and process issues, or suggestions for improving the supervision sessions, which the research team were also aware of. Their verbatim comments from the interviews are given below.

#### New skills

All the SC HVs mentioned how the training had given them new skills:

*‘It’s another string to my bow’ SC HV04*

*‘Being empowered to deliver CBT is just amazing. I don’t need to refer on and wait for someone to call back, I can do it myself. The skills it’s given me, it’s just immeasurable. I’m not mental health trained and I do really feel that I’ve learnt from it’ SC HV05*

The long waiting times for mental health support were mentioned by all the HVs, highlighting the reason for HVs to be trained to provide CBT-based support.

#### Relevance to health visiting

All four SC HVs thought that HVs were best placed to provide CBT-based support for parents and that all HVs should be offered the training:

*‘I think they [health visitors] should be given it in their training, because it’s something that actually works’ SC HV02*

When asked if HVs were best placed for CBT delivery, one SC HV replied:

*‘We’re here with them… We’re in the room with them anyway supporting them with their babies and their emotional health post-partum. So absolutely we are’ SC HV05*

*‘Just seeing it work and seeing the positive impact it’s had on most of my parents I’ve worked with has been so lovely and really lifted my spirits as a practitioner, to get that positive feedback that you really have helped somebody and that you’ve made a difference, and they haven’t had to wait a year for mental health support, it’s been really good’ SC HV04*

These findings were provided by just four HVs but are in keeping with the more complete quantitative and qualitative results from the initial SC study where 95% of 50 Regular HVs agreed that the SC materials were helpful for parents, 94% that they should be included in HV training and 86% that they were suitable for inclusion in the NHS (Bamber *et al.*, 2019).

## Discussion

This study’s purpose was to fulfil an interim role by developing a training module, confirming whether the SC materials were effective when delivered by HVs and preparing the way for a full-scale randomised controlled trial to compare the resulting SC-based service with services as usual.

The first objective was to work collaboratively with HVs to develop a training module based on the SC materials and tailored to fit their needs. With one proviso, this was successfully achieved. The iHV and senior HVs helped to develop the training structure and contents, including extensive use of online delivery. The SC HVs provided constructive feedback throughout their training, which helped to refine it.

The proviso is that the SC HVs were unable to benefit from the funding the study provided to the local authority for the time needed for recruitment of parents and SC HV training and delivery of the CBT-based sessions. Instead, the SC HVs had to add these to their busy working schedules, already disrupted by the Covid-19 pandemic. To mitigate this in future, the training has been modified in two ways. First, the contracts to commission the research and training will make explicit the time needed for the training and session delivery, and this will be agreed in advance with the administrative authority. Second, a discussion focusing on the challenges SC HVs are likely to encounter, and how to plan for them, has been added to the training. While these changes are unlikely to provide a complete solution, they should help to support the training in the future.

Our second objective, to assess whether it was feasible to train HVs in how to deliver the CBT-based support sessions and other materials and whether they could deliver them successfully under routine NHS conditions, was only partly achieved. Four of the 10 HVs who began the training did not complete it. However, the training and support sessions with parents coincided with the Covid-19 pandemic, which made parents ill and disrupted HV services and research projects. Looked at in this light, that 6 of 10 HVS were trained and delivered sessions in such challenging conditions was a triumph of professional commitment over adversity. Moreover, five of these six passed the ACCS assessment of CBT skills, while the assessors were confident that the one HV who completed the training but failed the ACCS would have passed given a second chance, which the training protocol allows. These findings demonstrate that HVs can be trained to deliver a service based on the SC CBT-based materials. The implication is that the practical arrangements needed to support SC HV involvement in research, while continuing to meet their professional responsibilities during a pandemic, were more of an obstacle to success than the challenges involved in gaining CBT skills.

Objective three was to assess whether the parents involved gained the same benefits as in the initial SC study and whether they approved the SC service. In view of the small numbers of parents and HVs involved, the results have been presented in relation to those from the initial SC study which included different groups of 52 parents and 50 HVs (Powell *et al.*, 2018; Bamber *et al.*, 2019). At baseline, 53% of parents in this study and 60% of parents in the initial SC study met the EPDS community screening criterion for probable depression. In both studies, too, the parents’ depression scores reduced substantially at outcome, after receiving the SC service. Although the mean outcome score in the two studies was similar, just 1 of 12 parents in this study continued to score above the criterion for depression at outcome. She was being treated by her GP doctor for chronic depression. In comparison, 29% of parents in the initial SC study retained above-normal depression scores at outcome. This may be because all the parents in this study received the CBT sessions, compared with half the parents in the initial SC study. Or, the larger group of parents in the initial SC study could make its EPDS outcome scores more representative. This can be examined in future studies, ideally including a routine-services control group and longer follow-up (Cooper *et al.*, 2003).

Using the GAD-7 scale, the majority of parents in this study were mildly, moderately or severely anxious at baseline, while their mean score fell below the threshold for mild anxiety at outcome. These findings and the improvements found in parents’ confidence, frustration and sleep were consistent with the initial SC study results in a larger and more complete dataset (Powell *et al.*, 2018; Bamber *et al.*, 2019).

Because parental frustration can trigger infant abuse (Barr *et al.*, 2006), the substantial reductions in parental frustration found are particularly important. Promisingly, too, all the parents involved in this study rated the SC materials highly and agreed they should be included in the NHS, mirroring the initial SC study findings (Bamber *et al.*, (2019)).

It needs to be kept in mind that the small number of parents in this study prevented tests of the statistical significance of findings. However, the consistencies in results across the two SC studies support their reliability and make it improbable that those from this study were due to chance.

The final objective was to provide the materials needed for a randomised controlled trial of the SC service and to consider whether that trial was warranted. The finding was that this study was successful as a pilot to tailor the SC CBT-based materials for delivery by HVs as part of NHS services. It has added, too, to the evidence that the service is wanted by parents and HVs and appears to support the wellbeing, coping and mental health of parents with excessively crying babies.

We conclude that a randomised controlled trial to compare the costs and effectiveness of the SC-based HV service with existing services is warranted, providing the practical impediments to the training and service delivery found here can be overcome. Supported by the NIHR HTA programme, a trial, including a pilot to demonstrate whether service delivery in diverse NHS settings is feasible, is underway (ISRCTN73761296, 2022). In combination, the long-term goal of the three SC studies is to support parent and infant wellbeing, help develop health visiting as an evidence-based profession and enhance the cost-effectiveness of NHS primary healthcare services. This study has contributed valuable evidence and lessons towards that goal.
